# Increase in Video Consultations During the COVID-19 Pandemic: Healthcare Professionals’ Perceptions about Their Implementation and Adequate Management

**DOI:** 10.3390/ijerph17145112

**Published:** 2020-07-15

**Authors:** Diana Jiménez-Rodríguez, Azucena Santillán García, Jesús Montoro Robles, María del Mar Rodríguez Salvador, Francisco José Muñoz Ronda, Oscar Arrogante

**Affiliations:** 1 Department of Nursing, Physiotherapy and Medicine, University of Almeria, 04120 Almeria, Spain; 2 Department of Cardiology, Burgos University Hospital, 09006 Burgos, Spain; ebevidencia@gmail.com; 3 Teaching Unit, Nursing Subcommittee, Primary Care District Poniente of Almeria, 04746 Almeria, Spain; jenrique.montoro.sspa@juntadeandalucia.es; 4 Knowledge and Research Management Department, Primary Care District of Almeria, 04007 Almeria, Spain; mariam.rodriguez.salvador.sspa@juntadeandalucia.es (M.d.M.R.S.); rondalia@gmail.com (F.J.M.R.); 5 University Centre of Health Sciences San Rafael, San Juan de Dios Foundation, Nebrija University, 28036 Madrid, Spain

**Keywords:** clinical skills, COVID-19, healthcare providers, implementation, interpersonal skills, perception, qualitative research, telemedicine, training, video consultation

## Abstract

In response to the COVID-19 pandemic, health care modalities such as video consultations have been rapidly developed to provide safe health care and to minimize the risk of spread. The purpose of our study is to explore Spanish healthcare professionals’ perceptions about the implementation of video consultations. Based on the testimonies of 53 professionals, different categories emerged related to the four identified themes: benefits of video consultations (for professionals, patients, and the health system, and compared to phone calls), negative aspects (inherent to new technologies and the risk of a perceived distancing from the professional), difficulties associated with the implementation of video consultations (technological difficulties, lack of technical skills and refusal to use video consultation among professionals and patients), and the need for training (technological, nontechnical, and social-emotional skills, and adaptation of technical skills). Additionally, the interviewees indicated that this new modality of health care may be extended to a broader variety of patients and clinical settings. Therefore, since video consultations are becoming more widespread, it would be advisable for health policies and systems to support this modality of health care, promoting their implementation and guaranteeing their operability, equal access and quality.

## 1. Introduction

Telemedicine is the use of telecommunication systems to provide health care from a distance [1]. This modality comes in different variations: online consultations, by telephone or videoconference, telemonitoring/screening with devices that monitor a patient’s vital signs, sensors with GPS trackers, and chatbots for recommendations [2]. However, the most commonly used are video consultations [1,3]. The main medical conditions that require video consultations are hypertension, diabetes, heart failure, asthma, chronic obstructive pulmonary disease and the care of elderly patients [3]. Therefore, video consultations have been widely used with patients who have common chronic conditions [4] and primary care needs [5]; these types of consultations are even considered the future of healthcare [6]. More specifically, nursing professionals have used video consultations in follow-up care for patients after surgery, patients with chronic diseases, families of children with cancer and premature newborns [7]. Telemedicine has demonstrated similar health outcomes and patient/healthcare professional satisfaction compared with in-person healthcare consultations, and has improved access to health care services [1,8]. In addition, some cost-utility and cost-effectiveness studies have demonstrated that telemedicine can reduce costs [1,8,9].

On March 11, 2020, the World Health Organization declared the novel coronavirus disease 2019 (COVID-19) outbreak as a pandemic. Most positive cases are asymptomatic or self-limiting, but the clinical spectrum of the disease extends to severe progressive pneumonia with acute respiratory distress syndrome (ARDS), which is a life-threatening condition requiring mechanical ventilation and intensive care support [10]. The symptomatology of COVID-19 infection is not specific, which makes it clinically indistinguishable from other viral respiratory illnesses [11]. According to recent systematic reviews, the most common disease-related symptoms are fever, cough, muscle aches and/or fatigue, dyspnea, headache, sore throat, and gastrointestinal symptoms [11] and skin lesions with different clinical characteristics [12]. In addition, there is evidence that COVID-19 may exacerbate certain cardiovascular symptoms and lead to cardiovascular complications [13]. Finally, some mental disorders have even been aggravated during this pandemic [14]. In this sense, nurses are central to COVID-19 prevention and the care of infected patients. Nurses are not only providing frontline care in severe COVID-19 cases that require hospitalization, but are also monitoring outpatients in community settings, and providing education to patients and the general public about the outbreak [15]. However, the rapid progression of COVID-19 around the world has become a real challenge for health organizations and policies, exemplified by the implementation of social distancing measures, such as quarantine periods [16]. As all healthcare professionals are at risk of contagion, new modalities of care are emerging in order to avoid face-to-face contact with patients and to ensure that patients receive the care they need [17,18]. Furthermore, as governments have been promoting social distancing, the creation of safe medical settings has become a priority [19] in order to avoid the risk of spreading the disease [20]. For instance, in Spain, 24.1% of positive COVID-19 cases have been reported among healthcare professionals [21].

In this sense, video consultations are considered the perfect solution during this worldwide pandemic [18], mitigating its impact on the population’s health and the use of health resources, and are being promoted, especially in the United Kingdom and the United States of America [22]. In addition, the population’s interest in telehealth and the number of telemedicine visits have dramatically increased during the pandemic, owing to restrictions on in-person clinical encounters [23,24]. Moreover, telemedicine platforms are ideal for responding to global infectious disease outbreaks and preventing overcrowding in emergency departments in hospitals, primary care clinics and emergency services [25]. Consequently, many governments have been forced to adapt to the sudden implementation of telemedicine to promote safety for low-acuity patients, their family members and healthcare professionals, and to avoid delays in the provision of health care that may result in more health problems or complicate existing clinical situations, jeopardizing patients’ future health [19]. For instance, online mental health services have been promoted within the context of COVID-19 [20]. Therefore, this pandemic is a call to action in countries without integrated telemedicine in their national health system [22].

In Spain, 100% of the population has access to the public health system. In response to the COVID-19 pandemic, the Spanish health authorities implemented follow-up systems at the primary care level, mainly consisting of phone calls [2]. In addition, some private health providers offered video consultations for the general public free of charge. Consequently, some Spanish health authorities are currently strengthening the use of teleconsultations and telemedicine as a public health policy for the reorganization and normalization of health care services.

However, studies have paid surprisingly little attention to the perception of telemedicine among healthcare professionals, mainly focusing rather on barriers to and difficulties with this modality of healthcare [3]. Thus, when a new approach is implemented, it is important to examine how it is perceived by healthcare professionals, as this could influence its usefulness and effectiveness. Furthermore, introducing video consultations to healthcare services is far more difficult than healthcare professionals assume, as they need to change their routines and the manner in which they care for patients [17]. Healthcare professionals need to understand that interactions during video consultations are different from face-to-face consultations, and thus, they need to be ready to deal with certain challenges: establishing a connection and starting a video consultation, dealing with disruption to the conversational flow, breakdowns of video consultation platforms and latency in the conversation [26]. Although at present health authorities are racing to implement virtual health-care technologies as fast as they can, and healthcare professionals are motivated to use them to reduce the spread of COVID-19 [27], their perceptions about this new modality of healthcare provision must be examined in order to ensure and improve its effectiveness.

For this reason, the purpose of our study is to explore Spanish healthcare professionals’ perceptions about the implementation of video consultations and its management in the provision of high-quality health care.

## 2. Materials and Methods

### 2.1. Study Design

A descriptive observational study was carried out using a qualitative methodology. This methodology is the most suited to achieving a deep understanding of the phenomenon of interest [28], such as the emerging implementation of a modality for the provision of health care that has an impact on the professionals who have to manage it. In this way, understanding their perception regarding video consultations may help us to understand this phenomenon and make more informed decisions regarding future courses of action. In addition, to ensure the quality and transparency of our research, The Consolidated Criteria for Reporting Qualitative Research (COREQ checklist proposed by Tong et al. [29]) was followed.

### 2.2. Sample and Setting

Although the sample size was not selected a priori, qualitative data were collected following two precepts: the data saturation precept proposed by Morse [30] (interviews continue until new elements of discourse are no longer collected) and the novelty precept proposed by Mayan [31] (data is collected until it is considered that something important and novel could be derived therefrom about the phenomenon of interest).

All study informants were working healthcare professionals in the public health system in the same Spanish region. Consequently, biases related to different organizational and care environments were avoided.

According to the recommendations for sample selection proposed by Patton [32], our purpose was to use selected participants (healthcare professionals with or without experience in video consultation) to obtain the greatest quantity of information in order to understand the phenomenon under study in depth. In this way, snowball or chain sampling was carried out, identifying relevant participants with the collaboration of key informants (training and research managers in healthcare departments) who nominated good candidates for our study.

Ultimately, the study included a total of 53 healthcare professionals. The study was carried out between 02 April 2020 and 25 May 2020.

### 2.3. Data Collection

A structured interview, comprising four closed-ended and four open-ended questions, was used to collect sociodemographic data and to facilitate in-depth discussion of all relevant topics (see the interview outline in Table 1). All participants were interviewed online (using the Google Meet^TM^ video platform (Google, Mountain View, CA, USA)) due to social distancing restrictions during the COVID-19 pandemic. The interviews lasted 15 min on average and were carried out by a researcher with extensive experience in qualitative research, who had no working relationship with any healthcare professional and strictly followed the interview outline, thus ensuring objectivity and avoiding possible biases. The entire content of the interviews was recorded with the participants’ consent.

### 2.4. Data Analysis

The mean and standard deviation (SD) were calculated to analyze sociodemographic data and responses to closed-ended questions. Qualitative data were obtained from the healthcare professionals’ responses to the four proposed open-ended questions. All perceptions and opinions from the healthcare professionals were transcribed and reviewed by two different members of the research team who were experts on qualitative methodologies. A content analysis of the qualitative data was performed; this allowed us to discover the views of each health professional by analyzing their responses and perceptions about the phenomenon of interest [33]. The themes identified were aligned with the four proposed open-ended questions.

Subsequently, qualitative data were analyzed to identify reiterated words, sentences, or ideas that were finally codified into different categories and grouped into the identified themes [31]. Firstly, an initial reading of the discourses was performed to analyze the categories. Then, the emerging categories were codified through the consensus and refinement reached by the two researchers [31].

All data were stored, managed, classified, and organized using the qualitative data analytical software, ATLAS.ti 8 Windows (Scientific Software Development GmbH, Berlin, Germany).

### 2.5. Ethical Considerations

This study was approved by the Research and Ethics Board of the Department of Nursing, Physiotherapy, and Medicine at the university (nº EFM 75/2020), and was carried out following the ethical principles for medical research of the international Declaration of Helsinki [34]. All participants received information about the study, participated voluntarily, and provided their written consent. In addition, and to ensure anonymity, the participants were numerically labeled in chronological order according to the date of the interview, preceded by the letter “S” (subject).

## 3. Results

The quantitative data collected from the closed-ended questions showed that 96.2% of the healthcare professionals considered videoconference consultations to be an adequate option for providing health care, indicating which patients would most benefit from this modality. The types of patients that were mentioned most often, i.e., in 14 to 7 informant responses, were chronic patients, patients who required medical follow-ups and examinations, difficulties in movement (either due to physical disability or geographical dispersion, or work reasons), and administrative petitions (such as prescriptions, work leaves due to illness or accident, etc.). However, the types of patients that were mentioned least often, i.e., in 4 to 2 responses, were resolutions of medical questions (from patients, caregivers, mothers, etc.), any type of medical condition that did not require physical examinations, on-demand consultations such as screening to evaluate in-person assistance or not, health education, mental health disorders, common, minor diseases, and dermatology. Furthermore, most interviewed professionals had not provided health care via videoconference (*n* = 44; 83%). Conversely, the number of video conferences among healthcare professionals who had used this modality ranged from 1 to 5 (mean = 2.66; SD = 1.322). Lastly, 90.6% of participants considered it necessary to train and educate professionals in this modality of healthcare. It should be noted that no differences were found based on gender or professional category in any quantitative data collected.

As for the qualitative data, results were obtained after analyzing the contents of the open-ended questions. During this content analysis, possible divergence in the participants’ discourse according to their professional category and workplace (primary care or hospital services) was taken into account, although these factors did not affect the majority of the categories identified, with a few exceptions, as described in the corresponding category, presented below and grouped into four main themes (aligned with the four open-ended questions) and the categories that emerged from the participants’ narratives and that were strongly supported by them (see also Table 2).

### 3.1. Theme 1. Benefits of Video Consultations

Within the context of the COVID-19 pandemic, where interviews were conducted, the clear benefit of avoiding spreading the disease was confirmed by healthcare professionals. In addition, they considered that video consultations may provide numerous benefits, with no significant differences according to their professional category or workplace. In this way, the categories that emerged, in order of repetition frequency, are as follows:

#### 3.1.1. Benefits of Video Consultations for Both Healthcare Professionals and Patients

Our healthcare professionals referred to avoiding movement that may be unnecessary (both for patients who do not have to visit to health centers in person and healthcare professionals who do not have to travel to patients’ homes). This benefit was repeated in the discourse of most healthcare professionals.

*“It avoids the patient’s travels to health centers”*

(S39)

*“You can solve their problems without having to travel and they see you, which is important for them”*

(S42)

*“To avoid the loss of working hours in patients when the medical consultation coincides with their working time, avoiding the time required to the movement from their works centers to the health centers and the waiting for their turn“*

(S7)

*“The management of time, of the patient and the professional, convenience, flexibility, less bureaucracy”*

(S43)

#### 3.1.2. Benefits for the Health System

This category includes benefits such as efficient consultations, avoiding agglomerations and waiting lists, quick resolutions of common, minor diseases, a decrease in workload at healthcare centers, and cost reduction. In this sense, our healthcare professionals perceived that the implementation of video consultations may have direct benefits for the health system.

*“It favors accessibility and immediacy, and serves as an efficient filter for in-person consultation”.*

(S2)

*“in specific situations, it can decrease the waitlist time”*

(S10)

*“(…) less crowds in waiting rooms, streamlining of banal diseases”*

(S11)

#### 3.1.3. Benefits of Video Consultations Compared to Phone Calls

The informants indicated benefits of video consultations compared to phone calls, which is currently the most used modality of telemedicine in Spain. In this sense, informants perceived as beneficial the possibility of assessing patients’ physical aspect, in contrast to phone consultations. Since the interaction is direct, contact with the patient is ensured, allowing both verbal and nonverbal communication to occur.

*“You can see the patient’s face (…) you can assess aspects of non-verbal communication”*

(S3)

*“I have provided care on the phone with information* via *Telematics, although I think that the video consultation goes beyond, because it allows you to assess the expression and transmit more to the patient, re-enforcing communication”*

(S21)

*“A faithful contact with the patient is maintained, dedicating the time needed without interruptions”*

(S49)

### 3.2. Theme 2. Negative Aspects

Two quite different categories emerged: the first was consistent among the interviewed professionals, while the second was almost anecdotal.

#### 3.2.1. Negative Aspects Inherent to New Technologies

Obviously, since it is a technology that does not require physical contact, there are some medical procedures that are impossible. Our healthcare professionals highlighted two main issues: the impossibility of physical examinations or procedural techniques during video consultations, and its management and/or technological difficulties, such as a lack of access for both professionals and patients (especially for the elderly). In this case, general practitioners mainly indicated the impossibility of performing physical examinations as a major drawback. Although our informants considered some negative aspects of this modality, they did not propose any solutions to address them.

*“There is a lack of examination if it was needed”*

(S38)

*“The elder population and those who are not so old, for them to have the tools necessary to conduct it, and the knowledge, and this is relevant to some professionals”*

(S43)

*“For some patients, it use could be complex. For those who are older, they need the necessary support to be able to use this means of communication”*

(S50)

#### 3.2.2. Risk of Perception of Distancing From Professional

Healthcare professionals were concerned that their patients may perceive the use of video consultations as a form of distancing from the health professional. In this sense, healthcare professionals were concerned that relationships with their patients may deteriorate and/or the internet connection required to hold a video consultation may create an environment of mistrust for patients.

*“The perception of some patients of distancing”*

(S33)

*“Mistrust in the use of technology, difficulties for older people, who do not have the devices, the relationship can be seen as more distant”*

(S34)

### 3.3. Theme 3. Difficulties in the Implementation of Video Consultations

The difficulties that emerged from the informants’ discourse were inherent to the use of new technologies, which may be unfamiliar or challenging. The needs to provide resources to healthcare professionals so that they could hold a video consultation, and the need to train and shape them for the adequate use of this new modality, were underlined.

#### 3.3.1. Technological Difficulties

In anticipation of the future implementation of this healthcare modality, technology was a re-emphasized issue, related to certain patients having access to the required resources and technological difficulties for both professionals and patients, with particular emphasis on the elderly.

*“Not all the patients, especially the older ones, have access to these technologies or they don’t know how to handle them”*

(S12)

*“Lack of use by the professionals and the older patients”*

(S20)

*“Computer problems and the older patients who do not know how they work”*

(S38)

#### 3.3.2. Lack of Technical Skills Among Professionals and Patients

Another difficulty for the implementation of this healthcare resource is the lack of technical suitability of both professionals and patients, mainly regarding the elderly.

*“It needs more time and adequate technical skills from both parties”*

(S2)

*“Perhaps at first, until the population is familiarized with this technique”*

(S26)

*“Difficulty of older people to adapt to this method”*

(S50)

#### 3.3.3. Refusal to Use Video Consultations by Healthcare Professionals and Patients

This issue emerged in a handful of the analyzed discourses. Our informants were concerned about the possible refusal to use this new modality by professionals and/or patients.

*“Technical difficulties. Rejection of specific patients/doctors to this type of distance care”*

(S7)

*“Mistrust, resistance from both parties towards the use of the technology”*

(S34)

### 3.4. Theme 4. Skills Needed to Hold a Video Consultation and the Need for Training

Within in this theme, technological skills appeared once again to be the main issue, while nontechnical and social-emotional skills were second. To a lesser extent, the need emerged to adapt the technical skills that are required for this modality.

#### 3.4.1. Technological Skill

This is related to the need to adequately manage the software or application used and the technological requirements.

*“Handling of telematics tools”*

(S20)

*“Correctly use the technology”*

(S29)

*“Use of the informatics tools or applications”*

(S34)

#### 3.4.2. Nontechnical and Social-Emotional Skills

Informants emphasized a wide variety of skills, since they were concerned that such skills may not be adequately managed using a modality without physical proximity. In this way, they indicated the following skills in order of importance: effective communication (the most repeated), empathy and patience, verbal and nonverbal language, skills required for structured and guided clinical interviews, assertiveness, and conflict resolution.

*“Communication skills (active listening, empathy, emotional support), motivation, creativity, conflict resolution, patience”*

(S35)

*“Active listening, communication and clinical interview skills, deferred conflict resolution, fomenting trust through this medium”*

(S48)

#### 3.4.3. Adaptation of Technical Skills

This is related to the need to find a substitute for physical contact. Although the interviewees raised this concern, they did not propose any solutions.

*“Probability of guiding a self-examination”*

(S16)

*“Assess clinical aspects that can replace the clinical examination in part”*

(S32)

## 4. Discussion

Our results show that even though 83% of the interviewed informants had not conducted a video consultation, they considered it to be an adequate option for health care (96.2%). Most of our participants had not used this modality because the most common form of telemedicine in Spain is the phone call, although the Spanish health system is currently encouraging the use of video consultation as a public health policy for the reorganization and normalization of healthcare services [2]. Taking into account that video consultations are currently considered a necessary tool, the present study was proposed to explore Spanish healthcare professionals’ perceptions about their future implementation and adequate management in this country; it is very important to gauge health care providers’ perceptions of this approach, given that the they are the ones who will conduct such consultations, and therefore, the quality of this modality will be dependent on them. These perceptions have not been investigated to date, although recent studies have focused on the general population, indicating a growing interest in telehealth, as it is a highly-demanded modality of health care during the pandemic [24].

In addition, the interviewed professionals indicated numerous clinical situations and diseases where video consultations may be used (for both chronic and acute disease conditions). In this sense, they are in agreement with the reviewed evidence, in that this technology could be useful in cases such as chronic diseases, medical follow-ups, and mental health or dermatology examinations [7,35]. However, the interviewed healthcare professionals extended video consultation use to almost all patients who had access to this technology, as also indicated by new research on this growing field [36,37,38].

Furthermore, as this modality of healthcare may be complex, the healthcare professionals interviewed considered that training was needed (90.6%). This is because the most-utilized modality of telehealth in Spain is still the phone call [2]. However, it should be taken into account that the implementation of an effective program in telemedicine takes time [18], so it is logical that training and education would be needed. In this sense, this finding is consistent with the study by Portnoy et al. [39], who stated that healthcare professionals may be trained and shaped to be “telefacilitators”.

Telemedicine has been shown to be an ideal response to the COVID-19 pandemic, with its use having been greatly extended in recent months [25]. This was shown by healthcare professionals themselves, who indicated the avoidance of infection and spread as a clear benefit of video consultations. Also, many other benefits for both patients and healthcare professionals were noted, e.g., avoiding travel, wider availability, its immediate nature, saving time, ease of use and consequent increased efficacy [40]. In this way, video consultations improve accessibility, and can be used to opportunely tend to urgent concerns. Regarding time-saving, Calton et al. [40] stated that video consultations saved “windshield time” for home-visiting general practitioners.

Additionally, the healthcare professionals interviewed considered that video consultations offered efficient screening for consultations, avoiding crowds and waiting lists, allowing for quick resolutions of common, minor diseases, and decreasing workloads and costs in healthcare centers, as shown by other studies [1,8,9,25].

In contrast, our informants considered as negative the improper use of the technology and the inability to perform physical examinations. However, there are current platforms, applications, and medical devices that may compensate for the need to perform such examination at patients’ homes, so medical procedures or techniques may be adapted to some patients [41,42]. In this sense, it is important to ensure that remote healthcare professionals are able to see that the patients are performing the examination correctly [41].

The difficulties identified by our informants regarding the implementation of video consultations were consistent with those of other studies [1,3,8,39,41]. Technological difficulties are the most worrisome issue among healthcare professionals. In addition, it should be noted that the refusal to use video consultations may be solved by performing preliminary trials, which often improve attitudes towards technology [40]. However, problems may arise among patients of advanced age, who may have reduced cognitive abilities [3]. Conversely, the barriers perceived by patients for the implementation of video consultations should also be taken into account. In this study, the perception of patients was not addressed. In this sense, a previous study indicated that although patients were willing to use them, they will likely go back to in-person consultations, as they may prefer to be attended to by their usual healthcare provider, or they may even ignore video consultations if they do not know how to use them [39]. There is no doubt that video consultations are needed in different health systems; therefore, this pandemic is a call to action in countries without such an option already integrated into their health systems [22]. However, most countries have not created a regulatory framework to authorize and integrate telemedicine into their national health systems, including during emergency and outbreak situations [22]. Although our informants did not indicate any ethical issues related to the use of video consultations, previous studies have raised concerns that exchanging health information and providing care electronically could create new risks regarding the quality of healthcare, safety, privacy and confidentiality [43,44]. As for the skills needed to hold a video consultation, the healthcare professionals perceived that they needed to be trained to improve their technological skills. This is congruent with other studies that identified the need for staffing qualified professionals in this modality of healthcare [45]. In addition, our informants indicated the need for training regarding both nontechnical and social-emotional skills, such as effective communication, empathy, patience, nonverbal and verbal language, skills required for a structured and guided clinical interview, assertiveness, and conflict resolution. They emphasized a wide variety of skills which they felt may not be adequately managed using this healthcare modality. Although social-emotional skills related to video consultations have not been analyzed in depth, Humphreys et al. [46] stated that the interaction between patients and health care providers during video consultation care was substantially different from in-person care, mainly in cases of palliative care or cancer patients. However, other studies indicated that both types of care may be similar if the internet connection is of high-quality [40,41]. In this way, patients and healthcare providers tend to communicate in the same manner as in in-person consultations. Minor technical breakdowns have been demonstrated not to cause major disruptions to clinical interactions [41]. In fact, video consultations have been considered an effective modality in the provision of health care to cancer and palliative patients and their relatives [40,47,48], for whom nontechnical and social-emotional skills are essential. Therefore, training regarding video consultation, to facilitate its adequate adaptation in the provision of high-quality care, is needed [46]. Furthermore, our informants perceived that they should be trained in adapting medical procedures and techniques, although this is more complex, as specific devices may sometimes be required at patients’ homes [41].

Video consultations were considered as a promising tool before the COVID-19 pandemic [49], and at present, are being used around the world [1,3] due to the need to avoid the spread of the virus [17,18]; therefore increased training and research in this field are required to ensure that high-quality health care is being provided. However, patients will also need to be provided with the devices required to adequately perform video consultations [41,44].

Lastly, as we carried out a small-scale qualitative study, there could be limitations related to the transferability of our findings. Nonetheless, this study aimed to address healthcare professionals’ perceptions about the immediate implementation of video consultations in their daily clinical practice, and this objective was achieved. In this sense, it should be noted that most interviewed informants had never held a video consultation, so their perceptions may change when they use this modality of health care. In addition, video consultations may differ according to the platform, software, or devices used. Consequently, more research on this topic is recommended. Lastly, it would be advisable to study the barriers perceived by patients related to the implementation of video consultations.

## 5. Conclusions

For the effective implementation of video consultations as a modality of health care within a health system, it is important to examine how it is perceived by healthcare professionals, as this could have an impact on its effectiveness. Our informants identified the positive and negative aspects related to video consultations, the difficulties associated with its implementation, and the skills required for its management; they also acknowledged that training is required. During the COVID-19 pandemic, the implementation of video consultations may yield information on the future of telemedicine with the goal of providing healthcare not only to chronic patients, but also to those with acute diseases. They have been shown to be useful in a broader sense, and so should not be stopped when the pandemic is mitigated. As our informants indicated, the use of video consultations may be extended to a wide variety of patients and clinical situations. It would be advisable to implement health policies and systems to support this modality of health care, promoting its implementation and guaranteeing its operability, equal access and quality of healthcare.

## Figures and Tables

**Table 1:** Interview outline.

Aspects Addressed	Questions
Sociodemographic data	Age
	Sex
	Professional category
	Work experience (years)
	Workplace (primary health or hospital services)
Closed-ended questions about general aspects of video consultations	Do you consider video consultations to be an appropriate means by which to provide health care?
	If your answer was affirmative, which patients do you consider would mainly benefit from this modality of health care?
	If you have already held a video consultation, indicate the approximate number of video consultations you have held.
	Do you consider that healthcare professionals need to be trained in this modality of health care?
Open-ended questions about specific aspects about video consultations	Indicate the benefits and positive aspects associated with the use of video consultations.
	Indicate the negative aspects associated with the use of video consultations.
	Indicate the difficulties in implementing video consultations.
	To properly hold a video consultation, which skills do you consider are relevant and should be taught?

**Table 2:** Comprehensive list of themes and categories identified after thematic analysis.

Healthcare Professionals’ Perceptions about Video Consultations’ Implementation	Theme 1.	Theme 2.	Theme 3.	Theme 4.
	Benefits of Video Consultations	Negative Aspects	Difficulties in the Implementation of Video Consultations	Skills Needed to Hold a Video Consultation and Training is Needed
**Categories**	Benefits of video consultations for both healthcare professionals and patients	Negative aspects inherent to new technologies	Technological difficulties	Technological skill
	Benefits for the health system	Risk of perceived distancing from professional	Lack of technical skills among professionals and patients	Nontechnical and social-emotional skills
	Benefits of video consultations compared to phone calls		Refusal to use video consultations by healthcare professionals and patients	Adaptation of technical skills
